# Elastic textile-based wearable modulation of musculoskeletal load: A comprehensive review of passive exosuits and resistance clothing

**DOI:** 10.1017/wtc.2025.2

**Published:** 2025-02-25

**Authors:** Amit Talukder, Jeyeon Jo

**Affiliations:** Department of Textiles, Merchandising, and Interiors, University of Georgia, Athens, GA, USA

**Keywords:** musculoskeletal load, exosuits, resistance clothing, biomechanics, elastic textile

## Abstract

Elastic textiles play a critical role in passive wearable solutions for musculoskeletal load management in both passive exosuits and resistance clothing. These textiles, based on their ability to stretch and retract, can exhibit ambivalence in their load-modulating effects when used in occupational, rehabilitation, exercise, or everyday activity settings. While passive exosuits and resistance garments may appear similar in design, they have opposing goals: to reduce the musculoskeletal load in the case of exosuits and to increase it in the case of resistance clothing. Despite this intrinsic connection, these two approaches have not been extensively linked together. This review aims to fill this gap by examining the common and distinct principles of elastic textiles in passive exosuits and resistance clothing, shedding light on their interactions and the complex dynamics of musculoskeletal load systems. The effectiveness of different designs in passive exosuits that mimic musculoskeletal function and resistance clothing that increase the workload for strength training are critically reviewed. Current challenges in practical implementation and opportunities to improve critical issues, such as preload, thermal comfort, skin friction, and donning and doffing are also highlighted.

## 1. Introduction

Throughout history, physical limitations of the human body have been overcome to gain more strength, move faster, and perform longer by using external tools (Lee et al., 2018). This desire has been expanded to the solutions for the rehabilitation of those with motion impairments (Rodríguez-Fernández et al., 2021). External or non-wearable tools, like vehicles, lifts, and medicines, certainly have their own benefits but have definite limitations in versatility, cost, portability, and flexibility. Wearable systems are thriving in human augmentation with the rise of flexible electronics and bionics. Often called exoskeletons or exosuits, these wearable robotic systems symbolize the integration of advanced technology with human physiology, offering enhanced strength, mobility, and endurance to the end users.

Exoskeletons and exosuits, although often grouped, refer to distinct types of wearable robotic systems. Exoskeletons use rigid structures parallel to the body connected at specific points to support and enhance body movements, providing added strength and stability (Asgari et al., 2022). In contrast, exosuits are cable-driven and pneumatic actuators based. Cable-driven exosuits connect the anchor points of the device via textile-based structures, making them lighter and portable and integrating more seamlessly with the body, aiming to support mobility without restricting natural movement. Pneumatic actuators-based exosuits use compressed air to power these actuators, similar to artificial muscles, which can provide an adaptable force application compared to exoskeletons. The newly developed applications using these technologies to adjust the loads on the human body are already actively being adopted and proving their capabilities in human performance and health care (Al-Fahaam et al., 2018). Researchers have developed various applications for construction workers, healthcare professionals, rehabilitation, sports training, and occupational health (Pérez Vidal et al., 2021). The military community has investigated and improved exosuits to increase the strength of soldiers (Crowell et al., 2019). It is mentionable that exosuits also assist people with disabilities and handicaps (Hsiao et al., 2019).

Both exoskeletons and exosuits can be further categorized into active and passive systems. Active systems rely on powered components, such as motors or actuators, to assist movement, while passive systems leverage the moments from the mechanical structure to reduce the musculoskeletal loads of the body using mechanical elements like springs or elastic bands, without external power. It is evident that the active system with powered electronics may generate a better outcome compared to the battery-free application solely relying on the materials’ passive properties (Quirk et al., 2024). A comparative study between the active and passive back exosuits revealed that the passive system decreased the back muscle activity by 13% (15% with an active system), but also added restrictions to waist flexion, unlike the active suit, which indicates the passive system’s nature requiring the human input to be activated (Quirk et al., 2024). Similarly, active back exosuits reduce back muscle loads by 25–41%, while passive suits reduce them by 16–18% (Poliero et al., 2022). Pneumatic actuators are another standard option creating active exosuits, using compressed air to create a movement that provides smooth, adjustable force (Al-Fahaam et al., 2018; Cuttilan et al., 2023).

However, considering the vulnerability of the human body and limited carrying capacity, active systems often with rigid, large, and heavy components may not always be the best option (Thalman et al., 2018). The usability and portability of the active exoskeletons are restricted due to their weight (13–48 kg) and rigid structure (Quintero et al., 2011; Kilicarslan et al., 2013). Ensuring proper alignment of the rigid robotic joints with the limb joints of the wearer is essential but often difficult because of the indistinct nature of the human joint center of rotation (COR; Jacquelin Perry, 2010). Misalignment may result in heightened resistance and discomfort, necessitating greater exertion and compensatory patterns of movement (Schiele, 2008). Self-aligning systems can tackle this challenge, but they also tend to increase the overall mass of the system (Stienen et al., 2009; Ergin and Patoglu, 2011). For example, weight placement near the foot and ankle will cause significant difficulties to the wearer’s mobility and the system’s energy consumption (Browning et al., 2007). Also, the maintenance of the active soft systems, including charging the battery, ensuring the waterproofness of the electronics, fixing issues in the complex mechanoelectrical structures, and securing the ownership and controllability of the digital systems, still remains a cumbersome factor that may make the public hesitant to accept the new highly functional technology (Bhatnagar et al., 2017; Christensen et al., 2021).

Passive systems without any additional power source, relying on the mechanical properties of the materials used, the human body energy, and sometimes gravity, can leverage the moments from the mechanical structure to reduce the musculoskeletal loads of the body (Ashta et al., 2023). While the idea of using passive force for human body actuation was introduced a while ago (Walsh et al., 2007), the interest in the role of elastic textiles as the primary contributor in the passive exosuit and their effectiveness has been increasing thanks to its softness and practicality in the real-life usage (Lamers and Zelik, 2021; Kowalczyk et al., 2023). The fact that most of the commercialized industrial exosuits are battery-free soft systems reflects their usefulness, affordability, and effectiveness in musculoskeletal load management (Krishnan et al., 2024. Passive systems offer unique advantages, such as simplicity, reduced weight, and the absence of a power source, making them ideal for endurance applications and scenarios where continuous support is needed without recharging. Especially, passive exosuits made of flexible, soft, and/or stretchable materials, such as textiles and elastomers have attracted attention by accommodating the needs for lightweight, comfort, cost-effectiveness, and coordinating performance (Koch and Font-Llagunes, 2021). Flexibility of the soft exosuits reduces limitations on the wearer’s movements, preventing difficulties associated with joint misalignment. Moreover, the suit with elastic components may exert torques on joints, potentially decreasing the number of actuators (Asbeck et al., 2013). These reasons lead to passive textile-based exosuits particularly useful in load lifting, rehabilitation, mobility assistance, and resistance training applications (Goršič et al., 2021; Chen et al., 2022).

While the passive exosuits are often designed to release mechanical strain and metabolic expenses, the passive nature of the system requires the wearer to provide the energy first to take advantage of the elastic potential energy (Washabaugh et al., 2021). This feature places elastic textile-based systems in a position to provide both load reduction and load addition functions. For example, an unpowered passive-elastic exoskeleton works alongside the knee and ankle joints during walking. It stores elastic energy in the spring when the knee extends at the end of the leg swing phase and then releases this energy to support ankle plantar flexion at the end of the stance phase, just before toe-off (Etenzi et al., 2020). This concept of elastic resistance has led to the development of wearable resistance clothing, incorporating elastic bands or cords directly onto garments to increase musculoskeletal load during movement (Almeida et al., 2017; Park et al., 2022). Elastic bands, such as Thera-Band^®^, provide an intensified training stimulus with less perceived effort compared to traditional weights, and their versatility makes them suitable for strength training, rehabilitation, and functional training for individuals with varied fitness levels (Almeida et al., 2017; Babiloni-Lopez et al., 2022; Lindberg, 2024). Elastic resistance clothing offers a convenient, user-friendly alternative for physical conditioning and is often integrated into shirts or pants; however, it may be less effective than traditional resistance equipment for joint support and high-intensity training (Torkildsen, 2023).

**Figure 1.**
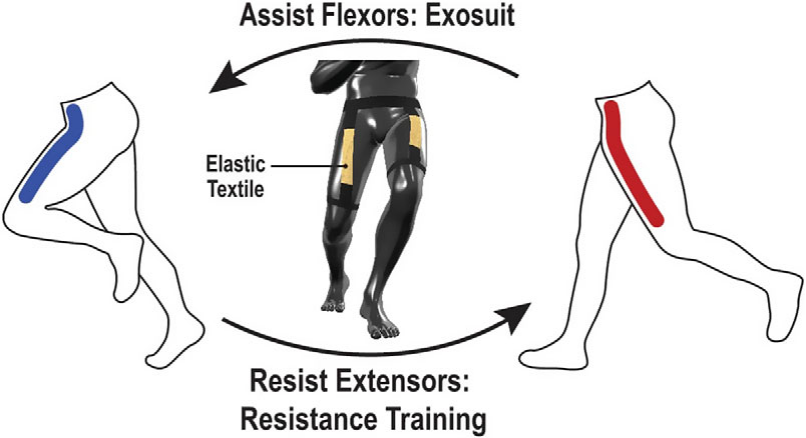
Elastic textiles that provide support and resistance for lower body movements.

The elastic textile-based passive exosuits and elastic resistance clothing, while their purpose is opposite – decreasing and increasing the musculoskeletal loads, respectively, often look and perform very similarly (Figure 1). Both have elastic components across the joints, and the human body stretches them. The retracting elastic potential encourages the body joints to go back to their original resting state (Liao et al., 2020). This is why wearable systems with elastic bands simultaneously assist and resist the human muscles (Krishnan et al., 2024. It emphasizes that any musculoskeletal load modulation system must be designed based on a deep comprehension of the human factors and the target task to achieve the desired enhanced/reduced muscle activity (Lamers et al., 2020). Ultimately, integrating elastic textile-based modulation of musculoskeletal stress via resistance clothing and passive exosuits can result in substantial advantages in physical performance, well-being, and safety of the wearer (Choi et al., 2020).

This narrative review aims to provide a comprehensive overview of the human body under a musculoskeletal load and how elastic textiles can assist in modulating the load in two wearable forms: passive exosuits and resistance clothing. Existing reviews often emphasize exoskeleton systems with active and passive components, soft exosuits with active components, soft exosuits for specific applications, or provide generalized coverage of wearable robotic technologies without delving deeply into the unique aspects of elastic textiles that both add and subtract load modulations to the musculoskeletal system (Gull et al., 2020; Jain and Jain, 2020; Koch and Font-Llagunes, 2021; Moeller et al., 2022; Zannat et al., 2023). Based on the interdisciplinary nature of the wearables, this review starts with the basics in biomechanics and textile science, hoping to offer a better understanding to roboticists, medical/exercise practitioners, and functional soft goods developers. The following sections introduce the current development status of the elastic textile-based passive exosuits and elastic resistance clothing applicable for the upper limbs, torso, and lower limbs. As mentioned earlier, the passive exosuit and resistance clothing can be very similar in terms of the elastic components crossing the joints. Therefore, this review differentiated the two based on the design goal of the product and/or the muscle activity results. If a system decreased muscle activation, we classified it as an exosuit, and if one aims to promote muscle exertion or growth, we classified it as resistance training. A discussion of the challenges and opportunities of the currently available technologies followed. By emphasizing the challenges and opportunities in innovative design approaches particular to passive, textile-based systems, this targeted approach closes a gap in the literature and offers a specialized viewpoint that enhances and expands on previous wearable technology assessments.

## 2. Methods

As a comprehensive review, this review started with a set of articles retrieved from scientific publication databases such as ScienceDirect, Web of Science, PubMed, and ProQuest, with the primary search string and keywords including but not limited to “passive” AND (“exoskeleton” OR “exosuit” OR “exoskeletal” OR “exotendon”) and (“resistance” AND “elastic” AND (“band” OR “clothing” OR “garment”)). Literature related to rigid exoskeleton assistive devices, non-soft robotic devices, conceptual soft robotics, manually operated exosuits, and exosuits that do not affect musculoskeletal loads was excluded during the screening process to solidify the focus on the passive exosuit and resistance garment. Additional articles related to elastic textiles, musculoskeletal structure, and adaptive clothing were reviewed to provide a complete and comprehensive narrative on this topic.

## 3. Musculoskeletal load

### 3.1. Biomechanics of musculoskeletal load

The biomechanics of musculoskeletal load is the study of physical stresses acting on and created within the human body and their effects on tissues and fluids (Hansen et al., 2006). The loads imposed on tissues, such as muscles may arouse physiological and psychological results, such as pain or discomfort. During voluntary or involuntary exertions and movements, muscles are essential for transmitting stresses through tendons, ligaments, and bones (Johnson et al., 1994b). Biomechanical models are frequently employed to estimate loading and its impact, because they impose issues while performing duties in the workspace, activities, and daily routines (Pitzen et al., 2002; Arjmand et al., 2009). Numerous internal and external factors, including body posture; exertions, forces; motions; environmental factors like thermal or vibrational energy transmission; and individual traits like anthropometry, strength, agility, and dexterity, all have an impact on biomechanical loading (Radwin and Lavenderand, 1999). Therefore, comprehending how these factors interact with the musculoskeletal system to affect internal loads on tissues, fluids, or muscles is important for enhancing performance, avoiding injuries, and supporting musculoskeletal health (Roman-Liu, 2013).

#### 3.1.1. Upper limb

**Figure 2.**
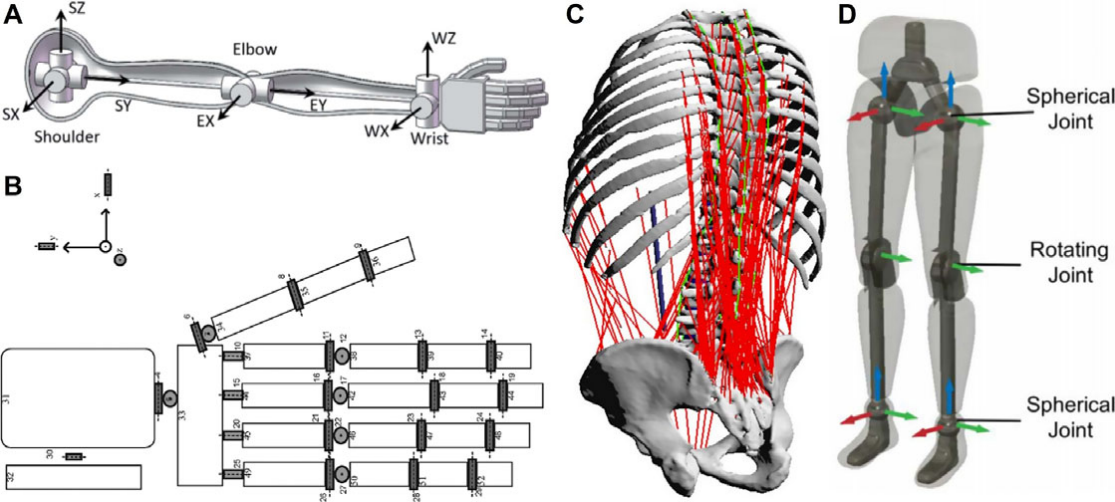
Musculoskeletal structure of the human body. (a) Arm bionics design with degree of freedom (Gong et al., 2019). (b) Hand segment model with rotational axis (Mirakhorlo et al., 2016). (c) Musculoskeletal load simulation on the human spine (Meszaros-Beller et al., 2023). (d) Lower limbs joint model with degree of freedom (Ren et al., 2020).

Comprising the shoulder, elbow, wrist, and fingers, the upper limb is a highly dynamic system essential for a wide range of functions of gross and fine motor tasks through coordinated muscle, tendon, ligament, and bone interactions (Betts et al., 2013; Pristerà et al., 2020). Each joint has specialized roles contributing to motor movement (Figure 2a). While the shoulder covers a vast area of motion, and the elbow performs as a solid lever, the wrist, and the fingers enable subtle and fine manipulations. Balanced muscle activation, efficient force transmission, and joint stability are essential for optimizing performance and reducing injury risk in the upper limbs (Georgarakis et al., 2022).

The shoulder consists of four distinct joints (glenohumeral, acromioclavicular, sternoclavicular, and scapulothoracic) that involve three bones: the clavicle (collarbone); scapula (shoulder blade); and humerus (upper arm bone) (Engín, 1980). The most mobile of the four joints, the glenohumeral joint, is essential to the upper body’s kinetic chain and allows for flexion, extension, abduction, adduction, internal/medial rotation, external/lateral rotation, and circumduction, three degree of freedom (DOF) in total (McLaren, 2023; Physiopedia, 2024b). it is crucial to acknowledge that the glenohumeral joint’s instantaneous COR shifts with upper limb motions and to consider it the dynamic COR while designing the shoulder mechanism of an exoskeleton (Stienen et al., 2009; Prinold et al., 2013). The elbow joint comprises the humeroradial and humeroulnar joints with 2 DOF, forming a synovial complex (Card and Lowe, 2024). The elbow enables forearm extension, flexion, supination, and pronation, with muscles like the biceps brachii, brachialis, brachioradialis, triceps brachii, and anconeus facilitating movement and stabilization (Card and Lowe, 2024). The wrist joint is a condyloid synovial joint that allows different movements: flexion/extension and adduction/abduction (Andrews and Youm, 1979). Wrist movements happen around an immediate COR and involve several muscle groups: flexors, extensors, and abductors/adductors, coordinating to ensure wrist dexterity (Neu et al., 2001; Bigale, 2024). The wrist joints are often susceptible to several injuries, including sprains, fractures, tendonitis, and dislocations (van der Sluis and Dekker, 2015).

Fingers are composed of phalanges and interconnected joints that play a vital role in fine motor skills and tasks requiring high precision. Each finger, except the thumb, has three phalanges connected by interphalangeal joints, enabling flexion/extension movements (Mirakhorlo et al., 2016). The metacarpophalangeal joints, where the fingers merge with the hand, additionally allow abduction/adduction to enhance the versatility of complex movements (Figure 2b). Fingers can deal with the forces necessary for daily tasks, such as grasping, typing, or manipulating small objects, which are often replicated in robotic grippers (Li et al., 2008). The flexor and extensor muscles, located on the forearm and the palm and connected to the finger phalanges by tendons, mainly coordinate to ensure precise control and stability (Schieber, 1995; Nimbarte et al., 2008). In addition, several intrinsic hand muscles, such as the hypothenar, interosseous, and lumbrical muscles, are located inside the hand. These muscles work together to provide fine motor control for accurate finger movements (Dominguez, 2018; Jones, 2023). Rehabilitation often aims to improve strength, dexterity, and joint mobility through specialized tasks with assistive devices (Klug et al., 2019).

#### 3.1.2. Torso

The torso consists of the spine, ribcage, and pelvis to support the body weight, protect vital organs, and facilitate movements as a central structure of the human body. The spine is an intricate structure made of 33 vertebrae, and it is categorized into cervical (neck), thoracic (chest), and lumbosacral (back and pelvis) regions (Cramer and Darby, 2014). Load distribution in the spine is up to movement patterns, posture, and external forces. Biomechanical models often analyze the impact of the forces on the spine during heavy load tasks and emphasize that maintaining a neutral spine position is critical to distribute the load evenly throughout the spine and reduce injury risk. The cervical spine’s primary role (the neck bones) is to support the head (Bogduk, 2016), typically weighing 2.3–5 kg. The cervical spine also ensures stability and supports head movements such as flexion/extension (pitch), lateral bending (roll), and rotation (yaw). The muscles around the neck control the movement and promote stabilization of the head (Johnson et al., 1994b). The thoracic spine provides stability and protection to the vital organs of the ribcage. Ribs that are connected to the thoracic spine create room and primary protection for organs and respiratory actions (el-Khoury and Whitten, 1993). The ribcage also serves as an attachment point for some upper body muscles, contributing to the stability of arms and shoulders and force generation (Lumb and Nunn, 1991; Romagnoli et al., 2006).

The lumbar spine plays a critical role in load bearing with the five largest vertebrae in the spine, offering 3-DOF: flexion/extension, lateral bending, and rotation (Hansen et al., 2006). Along with the core muscles, such as the erector spinae, rectus abdominis, obliques, and transverse abdominis, the lumbar region is responsible for movement balance, postural stability, and load distribution (Bogduk, 2016). Activities exerting loads on the spine, like repetitive bending, heavy lifting, or twisting, require a proper understanding and management of force distribution and body core capability to mitigate the lumbar injury risk (Hwang et al., 2021). 3D Simulations have been introduced to calculate the correct load distribution around the lumbar (Figure 2c). The pelvis, where the lower spine and the legs meet, transfer loads between the spine and the lower limbs (Pel et al., 2008). The biomechanical role of the pelvis is to balance stability and flexibility by bearing the body weights while supporting movements such as walking, running, and bending (Hammer et al., 2013). The pelvis rotates and tilts during gait to manage the generated moments and facilitate efficient movement (Hyung et al., 2016). Abnormal alignment in the pelvis can cause dysfunctional compensatory movement patterns and pain in the lower back and legs (Meuleman et al., 2011).

#### 3.1.3. Lower limb

The lower limbs consist of the hips, knees, ankles, and feet, accommodating body weight, supporting locomotion, and facilitating various essential human activities, such as jumping, walking, and running (Ren et al., 2020) (Figure 2d). The hip joint, as a ball-and-socket joint, allows for all 3 DOF in movements: flexion/extension, abduction/adduction, and rotation (Gold et al., 2024). Stability and mobility of the hip are maintained through the coordinated function of muscles, tendons, and ligaments. Key muscles include the gluteus maximus, which is responsible for extension, and the iliopsoas, a compound muscle that enables flexion (Martini et al., 2014). The gluteus medius and minimus on the lateral hip enable abduction and stabilize the body during gait (Sundstrup et al., 2014). The knee joint, primarily functioning as a hinge, is one of the most heavily loaded joints in the body (Nisell, 1985). It mainly allows for flexion and extension and a limited degree of rotation. The ligaments tightly hold the bones to keep them stable while guiding flexion and rotation (Gupton et al., 2024). Furthermore, the quadriceps (front thigh muscles) aid to stand up and straighten the leg, while the hamstrings (back thigh muscles) reaching from the hip to below the knee are essential for bending (He et al., 2007). The knee joint experiences significant forces during weight-bearing activities, so its capability to absorb and distribute the shock and loads effectively is essential for locomotion and musculoskeletal health, which assistive devices can contribute to (Machek et al., 2021).

The ankle has a hinged synovial joint enabling 3 DOF in movements: plantar flexion/dorsiflexion, abduction/adduction, and eversion/inversion, which is essential for basic gait patterns as well as the adaptability on various ground conditions and foot motions (Grimston et al., 1993). The muscles located in the lower leg drive the functionality of the ankle joint. For example, the gastrocnemius and soleus (calf muscles) can push the foot downward by contracting, whereas the tibialis anterior does the opposite (He et al., 2007). These muscles, ankle joints, and ligaments provide mobility, stability, adaptability to varied surfaces, and shock absorption (Houglum and Bertoti, 2011). The foot serves as the primary interface facing the ground and is the foundation of locomotion and weight-bearing (Manganaro et al., 2024). The three central regions – heel, mid, and forefoot – are specialized to facilitate gait, maintain balance, and absorb shock (Brukner, 2012; Physiopedia, 2024a). The intrinsic muscles of the foot provide fine motor skills and stability, such as toe movements and arch supports, while the extrinsic muscles originating from the lower leg control all significant foot motions. The arches mainly absorb shock but also function as a spring during gait; therefor, low arch (flat feet) or high arch can cause foot posture abnormalities, such as over/underpronation, leading to deficient gait. Foot orthotics are often the primary solution to redistribute forces and relieve the pain but they require customization to accommodate the unique foot shape of the individuals (Boudarham et al., 2014).

### 3.2. Muscle function and overuse

**Figure 3.**
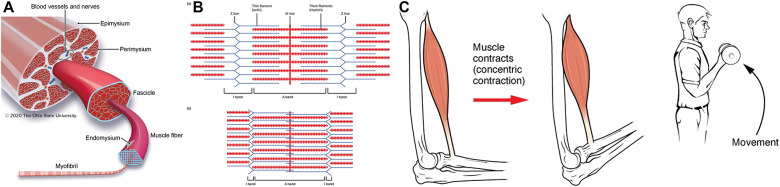
Muscle elements and contraction. (a) Structure of muscles (Jennings et al., 2020). (b) Muscle contraction mechanism (Rye et al., 2016). (c) Muscle contraction initiating the joint movement (Betts et al., 2013).

Muscle function is fundamental to human body movements and activities, which the muscle fibers coordinate to activate as the basic contractile units (Jennings et al., 2020) (Figure 3a). Muscle fiber contraction is the primary process to generate force and initiate joint movements. According to the sliding filament theory, two types of filaments within each fiber – actin and myosin – slide and get closer to the received neural signal, thereby shortening the muscle fiber (Rye et al., 2016) (Figure 3b). The collective shortening of muscle fibers results in the contraction of the whole muscle, which generates joint movements. For example, the long head of the biceps starts from the scapula (the shoulder blade), crosses the elbow joint, and reaches the forearm. When the biceps contract, the forearm is pulled toward the shoulder on the radius, triggering the elbow joint to flex (Betts et al., 2013) (Figure 3c). On the other hand, the triceps on the opposite side of the upper arm relax moderately during the elbow flexion to allow the biceps to function optimally and prevent excessive and unstable elbow movement (Seiber et al., 2009). While the muscle shortens during the concentric contractions, the muscles lengthen or maintain the same length during eccentric and isometric contractions. These contraction capabilities of muscles are vital in joint stability, protection, and body movement efficiency.

Muscle fatigue is a common phenomenon that occurs when intense use of muscles is done, leading to a decline in muscle performance. Intensive muscle activity causes a decline in performance, known as (Allen and Westerblad, 2001). Individuals often experience this muscle fatigue during activities like exercise, and it can also be present in various pathological conditions affecting the neurological, muscular, and cardiovascular systems (Enoka and Duchateau, 2008). Fatigue can arise due to a number of factors, including impaired neural activation, metabolic disturbances, and depletion of reserved energy. Muscle fatigue not only impairs physical performance but also increases the musculoskeletal injury risks. Muscular stiffness induced by fatigue may impair the capacity of muscles, including the paraspinal, to uphold stability, potentially heightening the likelihood of spinal instability and injury (Granata et al., 2004; Mawston, 2010). Comprehending how muscle fatigue affects musculoskeletal stability is crucial for developing efficient training regimes and supportive equipment, such as exoskeletons (Lamers et al., 2020).

Musculoskeletal injuries are frequently associated with heavy physical work, including repetitive lifting and forward-leaning, which causes recurrent load on muscles, tendons, ligaments, or joints (Luttmann et al., 2003). Repetitive strain without sufficient recovery, sudden high loads, or incorrect movement patterns can lead to overuse problems or acute injuries, such as sprains or strains. According to the US Labor Bureau, musculoskeletal disorders account for about 30% of work-related absences (Statistics, 2016). This issue is common across a variety of fields, including health care, construction, fishing, and agriculture (Smedley et al., 1995; Latza et al., 2002; Holmberg et al., 2003; De Kok et al., 2019; Rempel et al., 2021). For example, soldiers carry bulky loads in their daily tasks, which can easily exceed 31 kg (68.2 lbs) (Army Techniques Publication, 2017). Introducing these external loads will require the bearer to change their gait or posture or both to compensate. As both postural stability and gait mechanics can indicate a soldier’s health, determining how load carriage affects these postural stability and gait mechanics can be essential in preserving a soldier’s well-being. It is well-known that adding an external load to an individual will change both his kinematics (stride length, stride frequency, etc.) and postural stability, as that load will affect the location of the body’s center of mass (Seay, 2015). Therefore, forward lean happens to the individual’s body (Attwells et al., 2006; Birrell and Haslam, 2009; Seay et al., 2014; Nath et al., 2023a, 2023b). Besides forward trunk lean of the body, several factors, such as spine shape, spinal compression, spinal shearing forces, and thoracic–pelvic rhythm, have been associated with these load carriage (Attwells et al., 2006; Fowler et al., 2006; Meakin et al., 2008; Majumdar et al., 2010).

## 4. Elastic textiles

### 4.1. Material and fabrication

Throughout history, humans have used knitting, weaving, twisting, and stitching to decorate and warm themselves with textiles made from high-aspect-ratio fibers like cotton, linen, silk, and wool (Gries et al., 2016; Xiong et al., 2021). The twentieth-century need for functional textiles sped up the development of synthetic fibers like polyester, polypropylene, and polyurethane, as well as innovative production methods like thermal drawing, microfluidic spinning, and printing (Xiong and Lee, 2019; Dong et al., 2020; Zhang et al., 2020). Through creative approaches, including materials development, component integration, structural design, and fabrication processes, elastic textiles have been made possible by advances in materials and technologies. Elasticity is one of the major material properties that help materials recover from mechanical bending, stretching, twisting, and shearing (Liu et al., 2017; M. Park et al., 2012). Material or structure allows elastic textiles to deform and recover from external stimuli. They also accommodate and maintain deformations for specific configurations or circumstances (Hu et al., 2008; Levitt et al., 2020).

**Figure 4.**
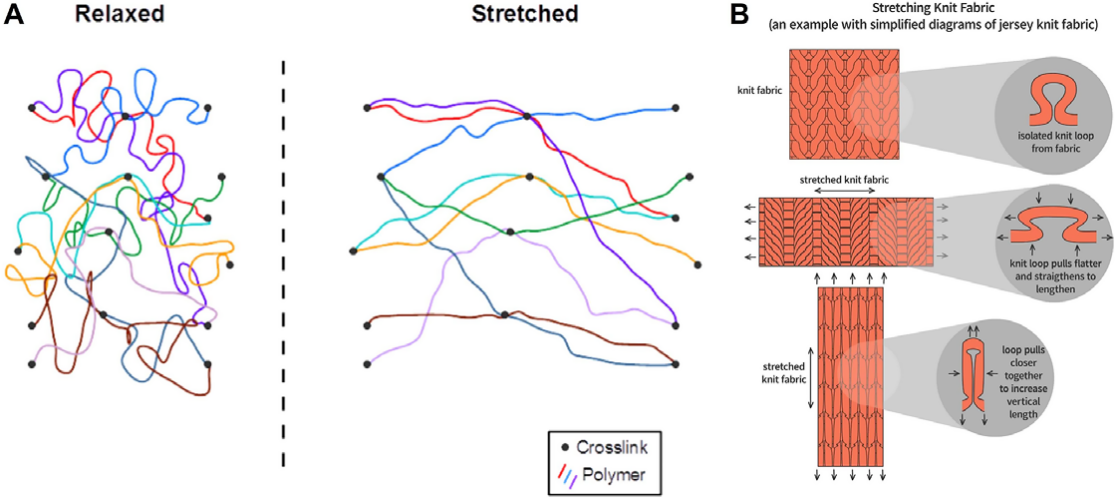
Elongation of fibers and textiles. (a) Elastomer under strain (Brzinski and Daniels, 2015). (b) Knitted textiles under strain (“The Why and How of Stretch Fabrics”, n.d.).

Elastic fibers can be made from synthetic and natural materials. Natural elastic fibers, such as rubber, utilize the malleable microstructures found in plants and animals (Wang et al., 2022). Conversely, synthetic elastic textiles generally depend on a deformable matrix and are prone to dynamic molecular displacement and reassembly. Elastic polymers are composed of a dynamic mixture of long, flexible, and rigid short chains (Hu et al., 2008). The extension of the flexible long chains happens by the axial displacement of the amorphous region (Brzinski and Daniels, 2015) (Figure 4a). The flexible amorphous chain region can relax once the external stressors are eliminated, allowing the material to recover. A long, flexible polyethylene glycol chain is reported to stretch up to 500 times its initial size (Hu et al., 2020; Zhang et al., 2021; Guo et al., 2022). One of the market’s most widely utilized elastic polymers is polyurethane (PU). PU is frequently utilized as an elastic matrix when creating functional elastic textiles, which are processed using conventional spinning and production methods such as wet spinning, thermal drawing, extrusion, casting, coating, templating, and printing (O. Akindoyo et al., 2016). These technologies improve elastic textiles’ utility and applicability by adding functional components. Popular elastic textiles such as latex and rubber elastic bands are used in fitness and rehabilitation workouts to offer resistance for strength training and muscle toning (Martins et al., 2013). These elastic bands come in various shapes, sizes, and resistance levels to assist users in achieving their fitness goals or rehabilitation (Lindberg, 2024).

Textile elasticity can be accomplished through the yarn structure forming the textile specified by the fabrication method. Textiles can be classified as woven, knitted, or nonwoven forms based on their manufacturing type. Knitted textiles, for example, can be elongated significantly due to the interlaced loop structure of the yarns (Sundstrup et al., 2014; Poincloux et al., 2018). The interconnected loops can withstand tension and contract when the elongating strain is released (Figure 4b). On the other hand, the warp-weft structure of woven fabrics tightly interlaced at the right angle does not allow much room to stretch compared to the knitted textiles. The nonwoven textiles, a web of fibers bonded mechanically, thermally, or chemically, sometimes allow for a limited range of elongation according to the fiber arrangements but often do not recover when the tension is removed (Yilmaz et al., 2020). Overall, incorporating elastic yarns into knitted and woven textiles is comparable, with careful consideration of yarn and fabric structure critical to defining the fabric’s elasticity and mechanical qualities (Huang et al., 2020; Zhang et al., 2022).

### 4.2. Mechanical properties in comparison to human musculoskeletal tissue

Material performance measurement is essential for ensuring safety, cost-effectiveness, and marketability. Textile elasticity is assessed through Young’s modulus, tensile strength, and elongation at break. Young’s modulus measures a textile’s stretchability and recovery under force; higher values indicate rigidity, while lower values suggest flexibility (Omnexus, 2024). Elastic textiles generally have lower Young’s modulus than other materials such as metals, ceramics, and glasses. For example, nylon has a Young’s modulus of 2.7 GPa, while glass fibers have 72 GPa (Omnexus, 2024). The tensile strength of elastic textiles defines the material’s ability to endure stretching forces without breaking (Hamburger, 1948). Textiles often show a linear-elastic behavior under tensile stress, and the deformation happens until it reaches its ultimate tensile strength (Rempel et al., 2021). Understanding this behavior is vital for designing textiles with optimal stretchability and durability. Finally, the elongation at break of elastic textiles means the amount of deformation under tension before failing, assisting in assessing stretchability and durability (Hamburger, 1948). Product developers incorporate elastane in textiles to increase the elongation capability. For example, the elongation at break was higher in textiles with a higher percentage of elastane fibers (Jovanović et al., 2022) (Ballard, 1995).

**Figure 5.**
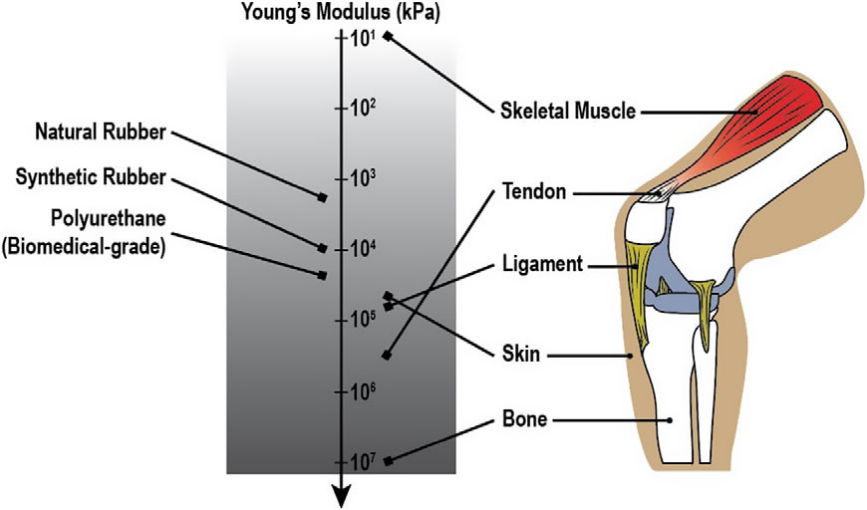
Young’s modulus of human muscle elements and elastic textile materials. The plot shows the average of the min–max value of the range described in Table 1.

When developing a wearable assistive system using elastic textiles, understanding the properties of both human body tissues and elastic materials is necessary to assign proper materials and guarantee the system’s performance (Lamers and Zelik, 2021). Figure 5 and Table 1 compare the mechanical properties of the commonly used materials for elastic textiles – natural/synthetic rubbers and biomedical-grade polyurethane – and various human tissues in the musculoskeletal system – skeletal muscle, tendon, ligament, bone, and skin – highlighting the difference in stretchability and strength.

**Table 1.** Mechanical properties of elastic textile materials and human muscle elements (Butler et al., 1984, 1986; Collinsworth et al., 2002; Devkota and Weinhold, 2003; Francois Francois, 2008; Garrett et al., 1988; Johnson et al., 1994a; Kuthe and Uddanwadiker, 2016; Mark, 2006; Martin et al., 2015; Ní Annaidh et al., 2012; Noyes and Grood, 1976; Takaza et al., 2013; Vermette, 2001; Vincentelli and Grigoroy, 1985; Vogel, 1987; Wood, 1977; Xiao et al., 2020)

Material	Young’s modulus (MPa)	Ultimate tensile strength (MPa)	Elongation at break (%)
Natural rubber	1–6 MPa	17–30 MPa	650–900%
Synthetic rubber	0.3–20 MPa	5–21 MPa	100–950%
Polyurethane (biomedical grade)	25–62 MPa	29–49 MPa	355–800%
Skeletal muscle	4.9–13.5 kPa	234–440 kPa	65–225%
Tendon	612–660 MPa	65–111 MPa	13–26%
Ligament	65–111 MPa	13–38 MPa	49–60%
Bone	10–17 GPa	51–133 MPa	2–3%
Skin	15–150 MPa	5–32 MPa	30–115%

Natural rubber and synthetic rubber display a high flexibility with Young’s modulus ranging 1–6 MPa and 0.3–20 MPa, respectively, while stretching significantly up to about 900% (Francois Francois, 2008; Mark, 2006; Wood, 1977). Considering these properties, rubbers can be ideal for products requiring both high resilience and elasticity. Biomedical-grade PU, meanwhile, shows a higher Young’s modulus (25–62 MPa) and textile strength (29–49 MPa), and comparable substantial stretchability up to 800% (Francois Francois, 2008; Vermette, 2001). Compared to the elastomers, the human tissues exhibit distinct differences. Skeletal muscle displays a much softer and more flexible nature with its much lower Young’s modulus (4.9–13.5 kPa) and tensile strength (234–440 kPa) (Collinsworth et al., 2002; Kuthe and Uddanwadiker, 2016; Xiao et al., 2020). It elongates up to 225%, which may be optimal and not necessarily extreme for body movements (Garrett et al., 1988; Takaza et al., 2013). In contrast, the tendon, connecting a skeletal muscle to a bone, and ligament, binding bone to bone, are much stronger, with Young’s modulus values of 612–660 MPa and 65–111 MPa, respectively (Noyes and Grood, 1976; Butler et al., 1984, 1986; Johnson et al., 1994a; Devkota and Weinhold, 2003). Their stretchability is also limited, up to 26% in the case of tendons and 60% for ligaments. The tendon and ligament’s properties reflect their role in joint stability while allowing some flexibility. Bone is the strongest and the most rigid among human tissues, displaying 10–17 GPa in Young’s modulus and a very low elongation capability (2–3%), showing its solid focus on the structural support (Vincentelli and Grigoroy, 1985; Martin et al., 2015). The skin exhibits a relatively wide range of Young’s modulus of 15–150 MPa as well as tensile strength (5–32 MPa) and elongates up to 115% (Vogel, 1987; Ní Annaidh et al., 2012). The skin covers the whole body, and its need to accommodate various needs, such as protection, movement, and growth, mirror the variability in the elastic properties.

The differences between elastic materials and human tissue shed light on the need for design considerations in wearable systems that mimic or complement the mechanical nature of the human body to provide necessary support without restricting biomechanical movement. Soft and wearable applications made of elastic textiles should enhance body performance while ensuring protection and comfort during demanding exercises, heavy load-bearing tasks, and repetitive bending. Balancing the rigidity to provide mechanical support and the flexibility not to hinder the joint movement is the key to design exoskeletons and resistance exercise outfits, which requires more research specialized in the target body area and motions on a deeper scale.

## 5. Elastic textile-based musculoskeletal load reduction: Passive exosuits

This section discusses elastic textile-based passive exosuits that minimize musculoskeletal load across body regions. We categorize these exosuits into three main types: upper limb, torso, and lower limb exosuits. Each design provides localized support to improve stability and reduce muscular strain during exercise.

### 5.1. Upper limb

The passive exosuit has shown vast potential in relieving upper limb movements in diverse conditions. These assistive devices can help with tasks in daily activities as well as in industrial and clinical settings. The upper limb exosuits could reduce muscle effort and support arm movements, benefiting individuals with motor impairments and musculoskeletal disorders. The arm muscles are one of the weakest muscles in the body and can become fatigued more frequently than the other larger muscle groups, such as the back and lower limbs (Chen, 2000). Localized fatigue in the upper arm muscles can cause lower back pain because the compensatory lifting posture may rely more on the back muscles, thereby increasing stress on the lower back. Therefore, assisting the arm muscles is recommended to prevent lower back injuries (Wan et al., 2022b). Reflecting the rapidly increasing needs for the upper limb assistance in various industrial sectors, such as manufacturing, construction, and shipping, a considerable amount of passive exoskeletons has been introduced both in the academia and the market (Moeller et al., 2022; Ashta et al., 2023). However, most of them utilize rigid structures made of metal or plastic to employ a solid exoskeletal lever and/or torque system (Balser et al., 2022; van Sluijs et al., 2024). Still, these rigid exoskeletons provided clues and opportunities to replace metal components, like springs and supports, with high elasticity and strength textiles.

**Figure 6.**
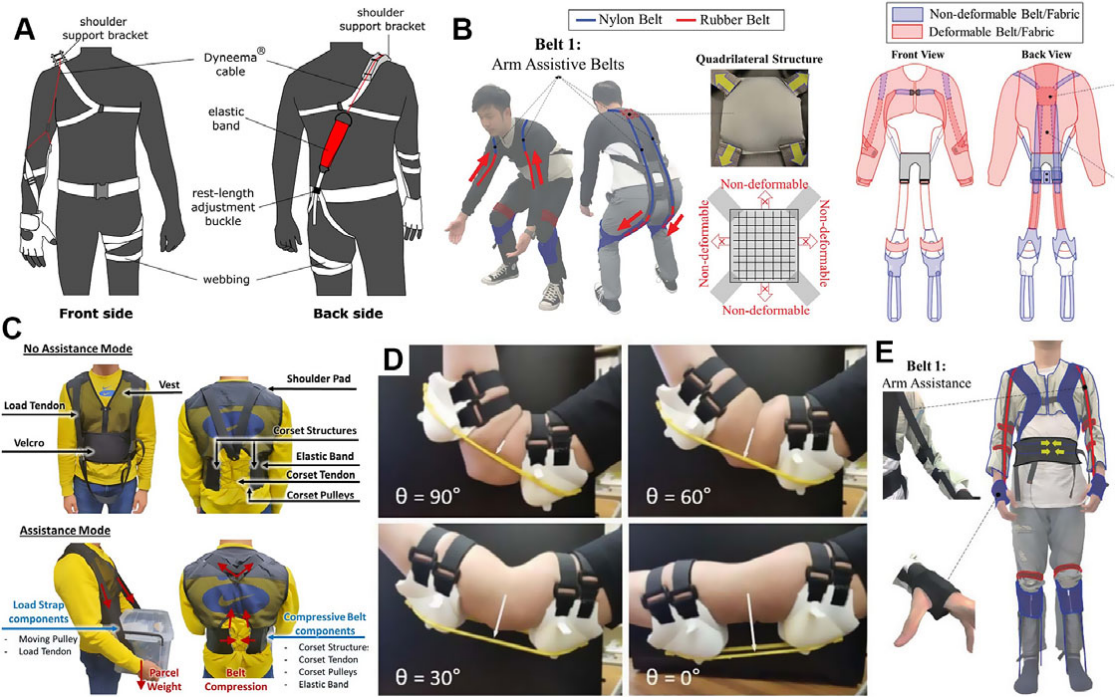
Upper limb passive exosuits using elastic materials. (a) Elastic band located on the back assisting shoulder movements (Joshi et al., 2022). (b) Full body assisting suit based on the combinations of deformable/non-deformable textiles (Liao et al., 2020). (c) Vest with a loading strap (Yoo et al., 2024). (d) Assisting elbow extension for poststroke patients (Phan et al., 2019). (e) Elastic band across the whole arm for arm movement assistance (Liao et al., 2020).

Joshi et al. (2022) developed a soft passive exosuit to assist the shoulder that primarily adopts elastic resistance on the back and an inextensible 2-mm-diameter cable reaching the elbow and crossing the shoulder (Figure 6a). The elastic resistance band, serving as a single exotendon in this suit, provides the elastic force, thereby reducing the shoulder load when the wearer raises their arm. In their experiments in shoulder flexion, the mainly contributing muscles, such as the deltoid and trapezius, showed significantly decreased muscle activity with the exosuit (Joshi et al., 2022). Liao et al. (2020) placed the elastic band directly in place of the biceps to assist the load-bearing tasks (Figure 6b). The full-body suit, including the shirt and anchors under the knee and at the sole, is called e.z.UP^®^ evenly distributes the heavy loads across the entire body, reducing the strain on the arm and back muscles (Liao et al., 2020). This was an updated version of their previous assistive upper body suit, which could support the arm muscles but not the back (Liao et al., 2018). Abs-Suit was designed for parcel delivery workers to reduce the increasing musculoskeletal disorders in the arms and the lower back (Yoo et al., 2024). It has a strong elastic band on the lower back that is connected to the load-bearing strap hanging around the front chest (Figure 6c). While the strap through which the delivery worker can place the heavy load is inextensible, the connected elastic band on the lower back provides distribution of the load and is configurable for the overall performance (Yoo et al., 2024).

An assistive device named PEMA (portable elbow movement assistant) was developed for stroke survivors, as some of them experience excessive muscle tone causing resistance to extend the elbow (Phan et al., 2019). The PEMA has elastic cords anchored on each side of the elbow to promote the elbow extension, as the elastic bands create recovering moments when the arm flexes (Figure 6d). The whole system mimics the musculoskeletal structure: while the elastic cords support the elbow extensor muscles, and the 3D-printed base frames, like a tendon, bind the cord to the arm. The PEMA significantly decreased the muscle activation of the triceps brachii, the main elbow extensor muscle, while making no changes in movement time, velocity, or biceps activation. In their another previous assistive exosuit, Liao et al. (2020) also reported a suit design where the elastic bands covers the whole arm joints, including the shoulder, elbow, and wrist (Figure 6e). They only referred to this as Prototype-3 without further details in terms of the arm support, but it showed the possibility of creating an undershirt soft exosuit that fully provides supports for all the muscles in the arm (Liao et al., 2020).

### 5.2. Torso

The torso, the core of the human body, is one of the most actively explored for opportunities of assistance in musculoskeletal load bearing (Ashta et al., 2023). This trend originated from the fact that 41% of workers report backache complaints (De Kok et al., 2019), and more than 370,000 incidents of back injuries resulted in missing workdays, according to the Bureau of Labor Statistics (Statistics, 2016). As a few occupations, including operators, fabricators, and laborers, account for more than half of back injury cases (NIOSH, 2004), the assistive solutions have been focused on the protection and support of the lumbar region of those performing heavy-lifting, load-carrying, and repetitive bending/twisting tasks.

**Figure 7.**
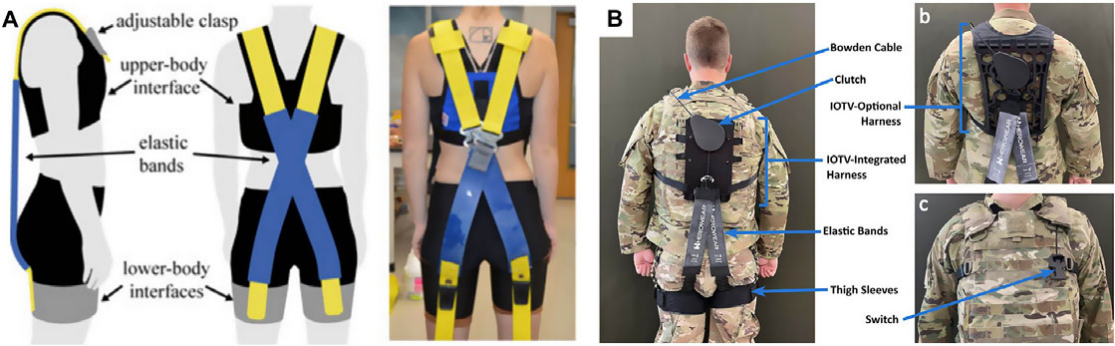
Exosuits assisting torso load management. (a) Low back load reducing suit (Lamers et al., 2017). (b) Soldier Assistive Bionic Exosuit for Resupply (SABER) prototype for US Army (Slaughter et al., 2023).

A widely adopted style combines the vest or straps for the upper body and the shorts for the lower body to create anchors around the shoulders, the waist, and the thighs, respectively. This lightweight, low-profile design mostly includes a strong and thick elastane crossing the back to evenly distribute pressure across the body, providing substantial support for tasks such as leaning and lifting (Figure 7a). When the user leaned forward or lifted objects, these bands elongated, exerting tension forces that ran parallel to the lumbar extensor muscles and ligaments. This motion resulted in a moment of extension around the lumbar spine, which relieved the lower back muscles and decreased muscle activity during tasks (Lamers et al., 2017, 2020). Another version of the same idea aimed to provide biomechanical assistance to the solders’ back and hip, which alleviates tension on the muscles and intervertebral discs (Goršič et al., 2021). This study showcased the effectiveness of an elastic, mode-switching back Exosuit in mitigating back pain and facilitating more straightforward lifting for soldiers during dynamic and realistic field operations (Slaughter et al., 2023) (Figure 7b). The high-friction elastomer on the inside of the thigh sleeves, along with a durable fabric on the outside, provided a secure attachment point for the elastic bands in the garment. Variable-length elastic bands connecting the upper body section provide the lumbar extension torque (13–16 N/m at 30° of trunk flexion and 17–24 N/m at 60° of trunk flexion) to help reduce strain on the user’s back muscles during forward bending. The researchers concluded that the surgical procedures in the medical field that generate significant exposure to trunk flexion angles of 20° or greater would benefit from this Exosuit by reducing muscle fatigue and discomfort (Kang and Mirka, 2023).

### 5.3. Lower limb

**Figure 8.**
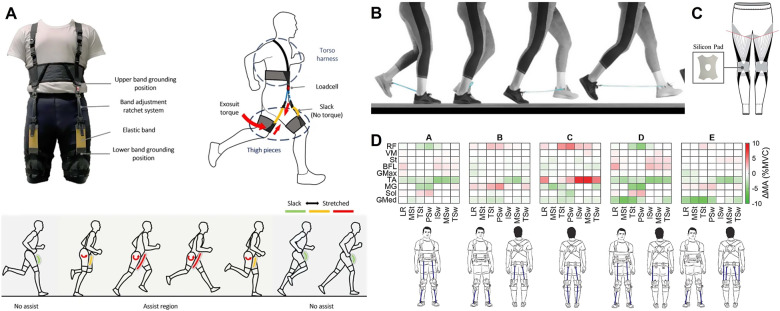
Lower body passive exosuits. (a) Elastic band assisting hip flexion (Yang et al., 2021). (b) Elastic cord connecting ankles to reduce energy cost (Simpson et al., 2019). (c) Two elastic bands on a knee assist sit-to-stand (Lee et al., 2020). (d) Configurations of the elastic band on the lower body (Krishnan et al., 2024).

Lower limb capability is essential in human locomotion and is critically aligned with the individual’s quality of life. Those with Parkinson’s disease, developmental disorders, or those impacted by stroke often experience gait issues and need external help to move from one place to another. Anchoring strong elastic bands at the waist and the right above the knee is a popular tactic to assist hip flexion during the gait (Schmidt et al., 2017; Panizzolo et al., 2019; Barazesh and Sharbafi, 2020; Yang et al., 2021; Kowalczyk et al., 2023). The elastic bands perform like a flexor or extensor muscle depending on the location. For example, the hip joint extension will stretch the elastic back on the front thigh, creating an elastic potential force promoting the leg flex back (Figure 8a). The force generated by the elastic bands can be adjusted by modifying the preload, and the anchor straps around the waist and thigh remain stable without causing unnecessary discomfort (Panizzolo et al., 2019). These elastic passive exosuits have contributed to correcting asymmetrical gait patterns and reducing metabolic costs (Panizzolo et al., 2019; Yang et al., 2021; Kowalczyk et al., 2023). A relatively unusual case would be a natural latex cord anchored at both sides of the shoes (Figure 8b). It creates moments whenever each leg flexes/extends, and the distance becomes longer than the cord’s relaxed lengths, eventually reducing energy consumption by 6.4% (Simpson et al., 2019).

X-tights with two embedded elastic bands cross a front knee to assist sit-to-stand maneuver (Figure 8c) (Lee et al., 2020). They motion-captured the skin deformation during the transition between the sitting and standing posture and installed the elastic band where the skin stretches most to get the maximized elastic energy return. Meanwhile, the potential of the elastic component in place of the plantar-flexor to decrease the soleus activation has been reported (Farris et al., 2013), Krishnan et al. (2024) examined different configurations to assist or resist selected muscle groups with the elastic bands, including ankle plantar flexion/dorsiflexion (Figure 8d Krishnan et al., 2024). In general, the elastic bands to assist hip extensors or ankle dorsiflexors decreased tibialis anterior activation, while the ones placed on the back showed varied results (Krishnan et al., 2024. It shows both the potential and limitation of the passive exosuit pants, as they decreased the overall muscle activation but could not accommodate all the opportunities to assist the metabolic cost at the individual phase of a gait.

Table 2 lists the applications introduced in this review based on the applications, materials, target regions, and effects. Decrease in muscle activation indicates that the muscle is exerting less effort to perform a particular activity while the exosuit is skillfully assisting the movement, thereby reducing the physical demands on the user, typically measured by EMG (electromyography).

**Table 2.** List of elastic textile-based assistive musculoskeletal modulation applications

Type	Materials	Weight	Targeted body regions	Applications	Decrease in muscle activation	References
Assistive	Nylon webbing, Dyneema^®^ rope, and Kaytan latex resistance band	600 g	Shoulder (Deltoid anterior, Deltoid mid, and Deltoid posterior, and trapezius scapula)	Manual labor or rehabilitation	50% Reduction (Deltoid anterior, Deltoid mid, and Deltoid posterior, and trapezius scapula)	Joshi et al., 2022
	Woven rubber belts and nylon belts	0.75 kg	Arms (biceps brachii) and back (pectoralis major)	High-load activities	34–47% (biceps brachii and back pectoralis major)	Liao et al., 2020
	Woven rubber belts and nylon belts	–	Arms (biceps brachii) and back (pectoralis major)	Manual handling task	41.1% (1st holding), 43.1% (lifting), and 26.4% (2nd holding)	Liao et al., 2018
	Latex elastic bands, nylon fabric with TPU layers, load-tendons and friction pads for grip	520 g	Arms (medial deltoid, anterior deltoid, biceps brachii, and flexor carpi radialis) Lower back (erector spinae)	Load carrying and holding tasks.	48% (Medial deltoid), 20% (Anterior deltoid), 78%(Biceps brachii), 83% (Flexor carpi radialis), and 0% (Erector spinae)	Yoo et al., 2024
	Rubber and nylon belts	–	Elbow joint (biceps brachii muscle)	Rehabilitation	–	Phan et al., 2019
	Elastic bands	2 kg	Lumbar muscles (erector spinae)	Activities that involve frequent bending and lifting	23–43% (leaning), and 14–16% (lifting)	Lamers et al., 2017
	Elastic bands	1.5 kg	Lumbar region	Delivery workers	36% (hip extensor muscles)	Lamers and Zelik, 2021
	Elastic bands	2 kg	Lumbar region	Activities that involve frequent bending and lifting	26–87% (hip extensor muscles)	Lamer et al., 2020
	Elastic bands	1.5 kg	Erector spinae, rectus abdominis, middle trapezius, and trunk	Rehabilitation	15% (erector spinae)	Goršič et al., 2021
	Elastic bands	1.2 kg	Lumbar spine and back muscles	Military/army field operations	–	Slaughter et al., 2023
	Elastic bands	1.5 kg	Lumbar muscles (erector spinae)	Surgical environment	21% (erector spinae)	Kang and Mirka, 2023
	Rubber bands	645 g	Hip joints	Older individuals	3.3 ± 3% (hip extensor muscles)	Panizzolo et al., 2019
	Rubber bands	609 g	Hip joint and thigh muscles	Runners	4.7 (hip extensor muscles)	Yang et al., 2021
	Elastic bands	–	Hip joints	Rehabilitation	–	Kowalczyk et al., 2023
	Latex rubber bands	–	Lower limb	Runners	–	Simpson et al., 2019
	Elastic bands	300 g	Knee joints	Elderly or those with lower limb impairments	3.2 ± 1.5% (rectus femoris, vastus lateralis, biceps femoris, semitendinosus, tibialis anterior, and lateral gastrocnemius)	H. Lee et al., 2020
	Neoprene, polyethylene (LDPE), non-latex silicone-based bands	–	Lower limbs	Rehabilitation	–	Krishnan et al., 2024
	Textile and foam, Silicone rubber bands	1780–1800 g	Thigh muscles, (hamstrings, gastrocnemius, vastus, soleus, gluteus maximus, hip flexor, and tibialis anterior)	Rehabilitation	40% (Hamstrings, Gluteus Maximus and Hip flexor)	Barazesh and Sharbafi, 2020
	Elastic straps and textile brace	–	Lower back muscles (multifidus, longissimus segment of erector spinae, latissimus dorsi, and rectus abdominis)	Musculoskeletal disorders	–	Bhardwaj et al, 2023
	Elastic materials and elastic textile	–	Lumber region	Manual handling tasks	10.6% for two-layer suits and 16.1% for three-layer suits.	Wan et al., 2022

## 6. Elastic textile-based musculoskeletal load addition: resistance clothing

This section explores the elastic textile-based resistance clothing designed to add musculoskeletal load. Focusing on three types—upper limb, torso, and lower limb resistance clothing—these garments incorporate elastic bands or compression fabrics to enhance muscle activation, stabilize joints, and increase exercise intensity. Each resistance clothing type offers targeted support, contributing to improved physical performance and injury prevention.

### 6.1. Upper limb

**Figure 9.**
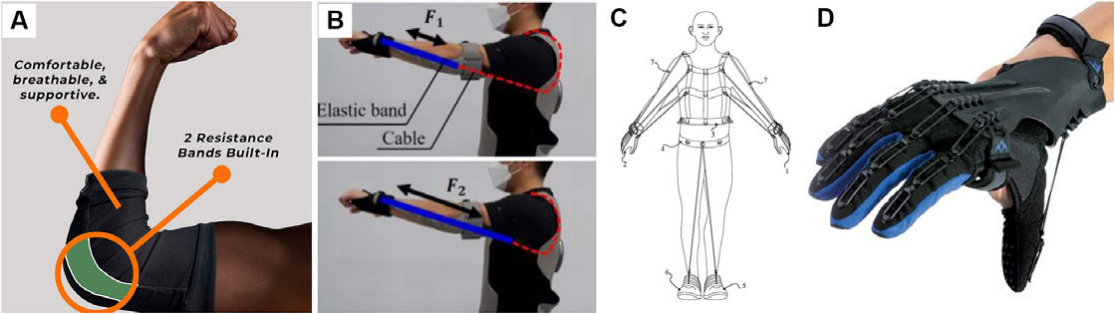
Upper limb resistance clothing. (a) Resistance elbow sleeve (“AGOGIE – Resistance Training Pants and Resistance Band Leggings”, n.d.). (b) Elastic band to increase the resistance to triceps (Park et al., 2022). (c) Multiple elastic cords connecting the gloves and the vest (Cranke, 2015). (d) SaeboGlove promoting the muscle growth for poststroke patients (“Hand Therapy Rehabilitation Glove for Stroke | SaeboGlove”, n.d.).

Resistance garments with embedded elastic bands can increase muscle use during exercise or daily activities. The elastic bands often flex the upper limbs so that the wearer activates the muscles to extend the arms or the other way (Figure 9a). A passive fitness wearable device with embedded elastic bands on the arms allows the wearer to simulate upper-body workouts, such as chest presses and frontal raises (Park et al., 2022). The elastic band connected to the glove extends up through the elbow and upper arm and is attached to a cable that adjusts the strength of the resistance force (Figure 9b). A similar and popular design to add elastic resistance is to connect the multiple resistance bands directly between the gloves and the vest with straps (Cranke, 2015; Felkel, 2015; Grove, 2021) (Figure 9c). The resistance bands were also placed between a pair of armbands and the side of the waistband (Cornish, 2017; Deinlein, 2018), wrist cuffs and shoes (Matthews, 2015), or arm-to-arm (Bergman et al., 2015; Alaniz, 2021). To prevent upper limb muscle atrophy during spaceflight, stretchy sleeves were designed for a neural posture in microgravity and anchored to the hands added at least 3 N to achieve full arm extension (Yilmaz and Goncu-Berk, 2022).

Most designs are reported as patents, so a limited number of studies evaluate the feasibility and effectiveness of resistance garments for upper limbs. The wearable fitness system with elastic band and cable, although it may not generate loads as high as gym equipment, resulted in a 1.5 times higher perceived exertion and twice the muscle activation compared to the bare body condition (Park et al., 2022). Similarly, a wearable resistance band harness increased total muscle thickness by 11% when the wearer was fully extended in the chest press position (Panizzolo et al., 2019). A resistance suit with two resistance bands attached at the elbow and the other side of the waist induced higher heart rates during push-ups and considerable discomfort in terms of tightness, scoring 2.83 out of 5 (Hansen et al., 2021). Hand orthosis with an embedded spring or tension cord to assist hand dexterity after stroke (Figure 9d) significantly enhanced grip strength (Barry et al., 2006), which virtual reality applications can facilitate further (Adams et al., 2019). Despite the limited assessments of the wearable systems, general elastic band-based resistance training protocols consistently showed a positive effect on muscle strength and athletic performance at similar levels to conventional methods (Lopes et al., 2019; Bauer et al., 2021). It is expected that the elastic bands incorporated into the garment system may display such an effect on the upper limbs.

Meanwhile, compression garments or sleeves without intended increased joint loading but their own stretchy textile did not significantly affect the physical performance of the upper extremities in general (Phan et al., 2019). However, when strategically designed, compression sleeves for the upper extremity demonstrated the ability to produce a torsional force on the forearm for hours. Supination, pronation, or no rotation was induced by the targeted design of the Lycra^®^ compression sleeves (Gracies et al., 1997). A study in which a compression splint immediately improved arm movements in supination and pronation in children with cerebral palsy also supports the idea of creating a load through elastic textiles (Elliott et al., 2011).

### 6.2. Torso

**Figure 10.**
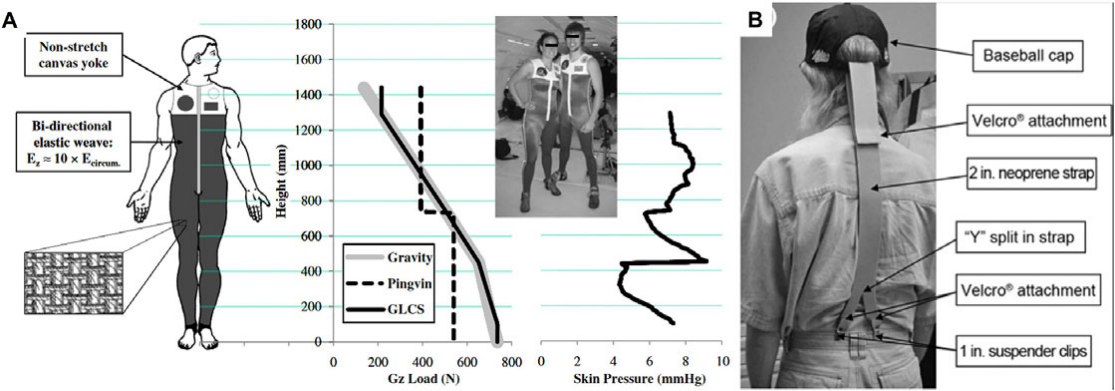
Elastic suits increasing the load on the spine. (a) Gravity-loading countermeasure suit for astronauts (Waldie and Newman, 2011). (b) Elastic head support for those with amyotrophic lateral sclerosis (Hansen et al., 2014).

Although passive exosuits with back supports aim to reduce lumbar load to increase work efficiency and decrease fatigue, resistance clothing seeks to enhance the body by adding loads that activate muscles or realign the spine. Prolonged missions in space have often caused spinal elongation in astronauts because of the low-gravity environment, which even increased in height (Zannat et al., 2023). Gravity-loading countermeasure suit was developed to add the axial load to the human body as much as on Earth (Waldie and Newman, 2011) (Figure 10a). This full-body suit is constructed of stretch/non-stretch weave or bungee cords to simulate upright loading across the body (Attias et al., 2017). It was effective at creating stress on the torso and spine, but it also caused significant discomfort at a level that the participants could not wear all day (Waldie and Newman, 2011; Breen et al., 2023).

Designed to align joints and strengthen muscle groups, especially for children with cerebral palsy, dynamic orthotic garments were inspired by astronauts’ resistance suits, because the motor pathology displayed a link to the negative effects of anti-gravity (Semenova, 1997). Rigid orthoses may provide better correction of spinal deformity and stability under gravity, but they have clear disadvantages in terms of discomfort and appearance, resulting in low patient acceptance (Wong et al., 2008). As in the microgravity system, dynamic orthotic suits made of elastic textiles apply loads to weak musculature and have a positive effect not only on facilitating muscle movements but also on balance (Semenova, 1997; Almeida et al., 2017). Suit therapy using dynamic orthosis like TheraTogs (TheraTogs Inc., Telluride, CO), in conjunction with proper exercise programs, reinforced the action of weak spinal muscles and stretched the tight tissues around the spine, thereby reducing kyphosis and improving range of motion in the thoracic area (El-Kafy and El-Shamy, 2022). Meanwhile, for those with weak neck extensors and difficulty controlling head drop, an elastic band between the back of the cap and the waistband or chest band can provide tension to support the head (Fast and Thomas, 2008; Hansen et al., 2014) (Figure 10b).

### 6.3. Lower limb

**Figure 11.**
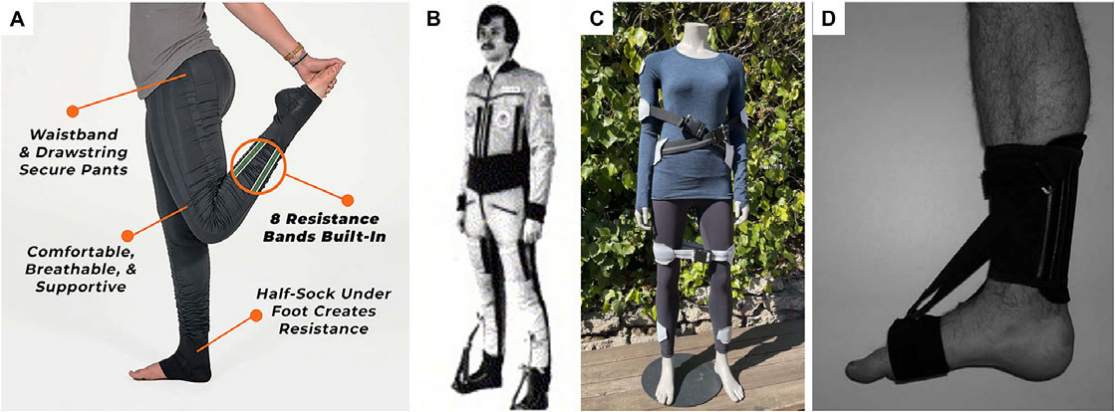
Resistance clothing for lower limbs. (a) Elastic band-embedded leggings (“AGOGIE – Resistance Training Pants and Resistance Band Leggings” n.d.). (b) Russian Pingvin gravity suit increasing the muscle load for astronauts (Artiles et al., 2016). (c) Elastic bands connecting the legs (Hansen et al., 2021). (d) foot-ankle orthoses further activating ankle muscles (Guillebastre et al., 2009).

Improving lower body mobility has been a major interest in resistance apparel. Elastic bands or cords often cross the legs and add loads during lower body movements to improve exercise quality and prevent muscle loss. A straightforward and popular design is to install elastic bands from the foot or ankle to the waist or torso so that the lower body bears the additional returning force of the elastic when the leg is fully extended or flexed (Benocci et al., 2015; Cranke, 2019; Allen et al., 2022) (Figure 11a). The Russian Pingvin (Penguin) suit included two bungee cords connecting the foot and waist/shin, respectively (Figure 11b). It stresses the lower body musculoskeletal system, up to 70% of body weight on the treadmill, thus preserving calf muscle and reducing lumbar bone loss (Artiles et al., 2016; Carvil et al., 2017). Another gravity-loading countermeasure suit also showed improved exercise performance in terms of blood lactate levels, initial ventilatory response, and perceived workload compared to regular gym clothing. (Attias et al., 2017). These effects of tension along the longitudinal axis of the lower body on exercise are confirmed in training sessions where lunges with an elastic tube looped from the foot to the shoulder induced high levels of hip, knee, and back muscle activity (Sundstrup et al., 2014).

A pair of elastic bands sometimes cross each other and connect one side of the thigh or pelvis to the other to increase loads during unilateral leg movements or to encourage postures that activate target muscles (Mial, 2015; Cornish, 2017; Stricker, 2020; Hansen et al., 2021) (Figure 11c). The effect of such designs on exercise performance has been reported in exercise studies using an elastic tube around the thigh, where using elastic bands improved the muscle activity of the hip external rotator during squats (Forman et al., 2023). Another study demonstrated that a 4-week training program using elastic tubing around the thigh not only significantly improved hip abduction force, but also the effect was also comparable to that of heavy resistance isotonic exercise (Folkins et al., 2021). Wearable tension across the legs also contributed to correcting squat posture. While the light resistance elastic band of 1.5 N/cm did decrease knee width index at various portions of countermovement jumps, reductions in knee width index occurred more frequently with the medium resistance band of 2.0 N/cm (Gooyers et al., 2012). Comparable results have been observed in research using a single resistance-level elastic band. Theraband^®^’s lightest band, 1.7 kg of resistance at 100% elongation, improved muscular activity in several lower limb muscles while maintaining an unaltered knee width index between conditions (Foley et al., 2017).

Elastic dynamic orthoses worn on the thigh, ankle, or foot have shown positive effects on the activation of relevant musculature in treating gait abnormalities and postural instability. TheraTogs, a dynamic orthosis, facilitated hip extensor and abductor muscle activity in an adult patient undergoing rehabilitation by applying pressure through elastic straps connecting the upper body and thigh (Maguire et al., 2016). Meanwhile, ankle–foot orthoses with an elastic band tensioned between the shank and foot (Figure 11d) improved gait abnormalities by increasing muscle activity, gait speed, and stride length, and alleviating foot equinus (Boudarham et al., 2014). Another study showed that this soft and dynamic orthosis can control plantar flexion without negatively affecting gait performance, unlike rigid orthoses that disturb gait velocity and step length (Guillebastre et al., 2009). The pressure applied by elastic ankle–foot orthoses also improved postural control by equalizing the weight bearing of the paretic and nonparetic limbs by activating muscle groups around the ankle (Kim et al., 2015).

Table 3 lists the applications introduced in this review based on resistive devices, their materials, target regions, and effects. Muscle activation increases by the resistive devices indicate that the exosuits offer resistance, necessitating more exertion from the wearer.

**Table 3.** List of elastic textile-based resistive musculoskeletal modulation applications

Type	Materials	Weight	Targeted body regions	Applications	Increase in muscle activation	References
Resistive	Four-way stretchy knit and non-stretchy woven fabrics	–	Elbow, hip, shoulder, and knee joints	Exercise in microgravity	–	Yilmaz and Goncu-Berk, 2022
	Elastic bands	1.3 kg	Upper limbs	Exercise	More than 100%, pectoralis major (clavicular head (CH), and sternal head (SH))	Park et al., 2022
	Elastic bands	–	Upper limbs	Exercise	Muscle thickness increased by 11% (Anterior, central, and posterior)	Owen et al., 2019
	Elastic materials	–	Thoracic Spine	Children with cerebral palsy	Improved postural balance	El-Kafy and El-Shamy, 2022
	Elastic bands	–	Entire body	Astronauts	Increased strain on torso, thigh, and shank	Waldie and Newman, 2011
	Elastic bands	–	Hip joints	Rehabilitation	–	Maguire et al., 2016
	Elastic bands	–	Ankle	Patients requiring orthotic support	–	Guillebastre et al., 2009
	Elastic bands	–	Knee Joints	Exercise and rehabilitation	–	Gooyers et al., 2012
	Elastic bands	–	Shoulder external rotators, elbow flexors, and hip abductors	Untrained college-aged females	Increased force production	Folkins et al., 2021
	Elastic bands	360 g	Entire body	Astronauts	Increased initial exercise ventilatory responses	Attias et al., 2017
	Elastic bungee cords	–	Entire body	Astronauts	–	Artiles et al., 2016
	Spring	–	Hand	Hand rehabilitation	Increased grip strength	Barry et al., 2006

## 7. Discussion: challenges and opportunities

The current review encompasses elastic textile-based passive applications to modulate the musculoskeletal load of the body with a purpose either to assist or resist. While the potential of wearable technology to mitigate musculoskeletal stress and injury risk is promising, understanding the functionality of textiles in terms of elasticity and accommodations for electronics is essential in developing soft wearable systems. The capabilities of active devices exceed unpowered ones, while their limitations in weight, comfort, safety, and maintenance led the researchers and the market to explore passive exosuits or resistance clothing. Passive systems clearly have limitations to overcome (Table 4); they cannot adapt to different energy-exerting styles of each individual or the phase of a motion. For example, in the performance test of an upper limb and the lower back assistive suit, males adopted different tactics to manage heavy loads – younger males used their arms, while middle-aged males tended to use lower-back muscles (Liao et al., 2020). Although most passive systems have adjustment capabilities, they cannot accommodate every individual habit or intermittent posture change. As the mechanical properties related to strength and elasticity affect the system’s performance (Yoo et al., 2024), the elasticity-based musculoskeletal load modulation system has to allow easy replacement of the elastic component to provide optimized assistance to the individuals.

**Table 4.** A summary of disadvantages of passive systems and potential solutions

Disadvantages	Potential solutions	References
Cannot accommodate every individual habit or intermittent posture changes	Adjustable elastic bands, personalized fitting mechanisms, such as customizable straps or adjustable anchoring points, Easy replacements	Yoo et al., 2024
Neutralizes the load modulation effect	Controlled resistance features by using multilayered elastic bands	Liao et al., 2020; Krishnan et al., 2024
Joint discomfort because of pretension	Sequential activation of multiple elastic components	Liao et al., 2020; Wan et al., 2022b
Sweat and skin heat production leads to thermal discomfort	Incorporation of sweat-reducing materials, in conjunction with easily accessible loosening/tightening interfaces	Elstub et al., 2021
Too thin or narrow elastic band leads to friction burns	Broader straps	Thalman et al., 2018
Skin friction	Using padded materials under the anchor straps and loading localized pressures	Awad et al., 2020; Kozinc et al., 2021; Yandell et al., 2020
Donning and doffing time issues due to a series of adjustable straps and buckles	Reducing straps and buckles	Cuttilan et al., 2023
Unwanted friction because of tight-fitted Exosuits and Resistance clothing	Design with a widened neckline and less compressive	Imamura et al., 2011

The ambivalence of the elasticity-based passive system – stretching and retracting – sometimes neutralizes the load modulation effect or even generates unexpected opposite results. For example, even if wearing passive exosuits to reduce muscle activity, the participants reported increased muscle activation during some postures because the system could not consider all the phases of the target activity (Liao et al., 2020). This neutralizing effect may impact on the overall work performance and metabolic energy consumption. Nevertheless, a significant portion of the articles reviewed in this study only reported the muscle activity of the target muscles, while there may be an opposite effect among the other muscles. The development of passive exosuits and resistance garments and their reporting should thoroughly examine the activity of all relevant muscles, not just a few muscles that perform a specific movement, to encompass the potential side effects of the system and achieve the goal.

**Figure 12.**
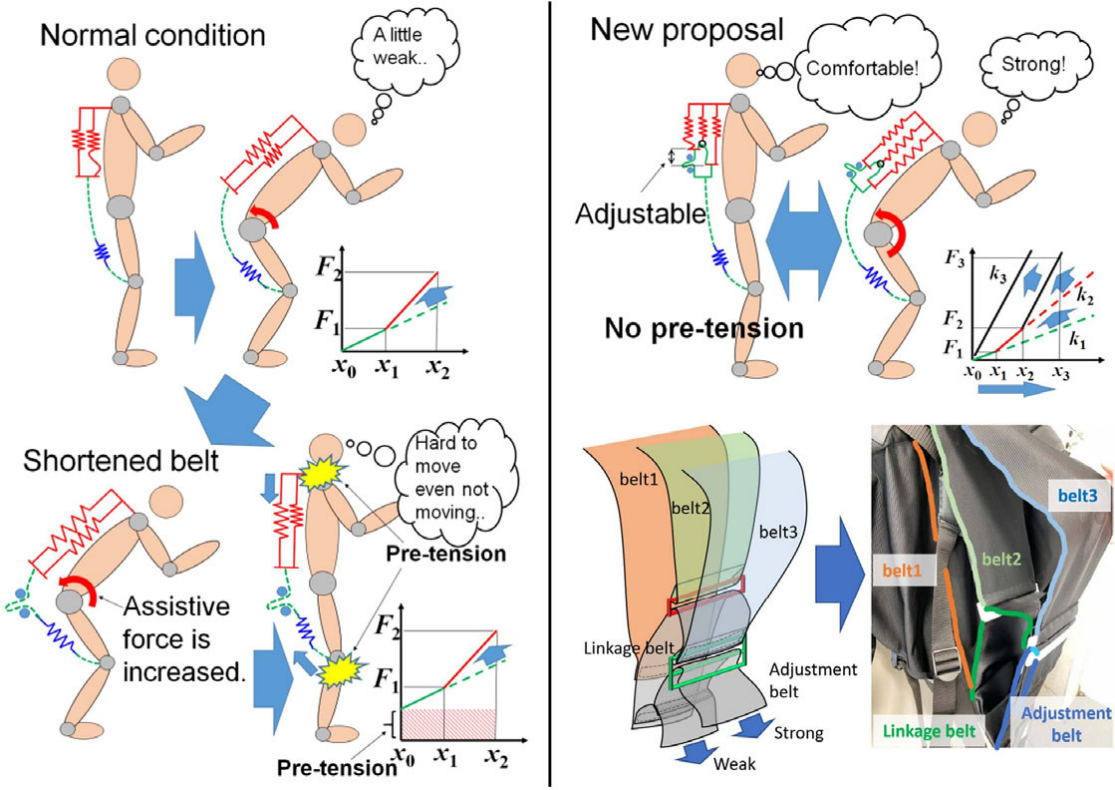
Layered elastic textiles to minimize the uncomfortable pretension (Wan et al., 2022b).

Pretension in elasticity-based systems is also a significant issue that needs to be broken through. The heavy load-bearing tasks require a quite high tension for the wearer to get substantial assistance, yet it generates perceived restrictions and decreased range of motion when unnecessary (Wan et al., 2022a; Quirk et al., 2024). Joint discomfort can arise from both highly tensioned deformable elements and non-deformable parts, as pretension makes the whole system tightly fit to the body (Liao et al., 2020). The postural discomfort from the pretension has been one of the most common reasons why people, often those in rehabilitation, gave up getting further assistance from the wearable systems (Hansen et al., 2014). Most importantly, this condition is undesirable because the discomfort from the tension loads and inconvenience at joints might introduce another compensatory posture or movement, causing another musculoskeletal disorder (Cho et al., 2018). A potential solution could be a sequential activation of the multiple elastic components (Wan et al., 2022b). In a three-layer rubber belt structure for the back support, there is no or minimum tension when the user is stranding and inactive, but the assistive tension rises when the user is bent as the number of involved elastic bands increases (Figure 12).

Thermal comfort is one of the biggest priorities in textile-based systems, depending on the thermodynamics of the environment and human thermoregulation (Del Ferraro et al., 2020). Elastic textile-based wearables often face challenges in maintaining thermal comfort because the lack of space between the skin and the textile interface causes heat retention, reducing ventilation and sweat evaporation (Gagge et al., 1969). During the human experiments with a passive exosuits, although the metabolic heat production has decreased, the participants showed a greater mean skin temperature and thermal sensation than the condition without the exosuit (Liu et al., 2021). The users of the passive exosuits may be able to perform longer, which will cause more sweat and skin heat production (Buono et al., 2010). The design improvement should count the varied environmental conditions with a more diversified participant group (Elstub et al., 2021) because differences in body size, shape, and muscle fiber composition can be responsible for the thermal performance variability (Lamers and Zelik, 2021). Less skin coverage can also aid thermal comfort management, but it should be considered that too thin or narrow elastic bands might cause friction burns or generate insufficient force (Statistics, 2016), and broader straps can improve the device’s comfort primarily when operating at elevated pressures (Thalman et al., 2018). Sweat-reducing materials, in conjunction with accessible loosening/tightening interfaces, may provide better thermal comfort for users (Elstub et al., 2021).

Skin friction is another major problem users experience (Awad et al., 2020). Like many compression garments designed without considerations about skin and muscle interactions (Weakley et al., 2022), when passive exosuits or resistance clothing does not reflect the human factors involved in the target activity, they can create unwanted frictions because most are tightly worn on the body. Imamura et al. (2011) found that tight fit and skin rubbing decide the comfort level of the wearers of a soft exosuit, called Smart Suit Lite. Their updated design with a widened neckline and less compressive but more stretchy vest significantly improved the wear comfort and load alleviation (Imamura et al., 2011). The abrasions on the skin can be softened by using padded materials under the anchor straps, and loading localized pressures (Yandell et al., 2020; Kozinc et al., 2021). The exosuits or resistance clothing design should enhance the user performance while not impeding regular movements or inducing unnecessary rubbings (d’Elia et al., 2017).

Donning and doffing have been reported as a critical factor in adopting and utilizing passive exosuits. The assistive wearable devices sometimes require someone other than the wearer to don and doff fully, but the users want to put them on independently or use only one hand (Krishnan et al., 2024. During the donning and doffing process, securing or releasing a strong elastic band is the most challenging part (Krishnan et al., 2024, which is expected for other systems with elastic bands, especially for elderly individuals or those undergoing rehabilitation. A series of adjustable straps and buckles may help the tension control, but they can extend the donning and doffing time, which impedes the rapid removal of the suit in emergencies (Cuttilan et al., 2023). This could also be related to productivity in the workplace or toileting issues.

## 8. Conclusion

Elastic textiles are critical in passive wearable solutions for musculoskeletal load management in passive exosuits and resistance clothing as applications in construction, health care, rehabilitation, sports training, military, and people with disabilities and handicaps. While passive exosuits and resistance garments may appear similar in design, they have opposing goals: to reduce musculoskeletal load in exosuits and to increase it in the resistance garments. While the properties of elastic textiles to stretch and retract may be closely related to the ambivalence in their load-modulating effects, existing reviews often provide only generalized coverage of wearable robotic technologies in the larger picture of active/passive exoskeletons and exosuits. This narrative review aims to fill this gap by providing a comprehensive overview of the human body under musculoskeletal load and how elastic textiles can assist in load modulation in two wearable forms: passive exosuits and resistance clothing.

The effectiveness of different designs in passive exosuits that mimic the musculoskeletal mechanism and resistance garments that increase the workload for strength training are critically reviewed. The ambivalence of elasticity-based passive systems with stretching and retracting behaviors can neutralize the load modulation effect or even produce unexpected opposite results, which require future developments and studies on passive exosuits and resistance garments to examine all relevant muscles and side effects of the system to achieve the goal in physical performance and metabolic energy consumption. The current review also identified common challenges in the practical implementation of elastic textile-based passive systems, such as preload, thermal comfort, skin friction, and donning and doffing. These challenges can be opportunities for future studies in addressing them and improving the overall system with innovative design approaches. Almost all textiles used in clothing have some degree of elasticity and create mechanical interactions with the human body during movement. The load exchange between the garment system and the wearer should be better understood and engineered to maximize human comfort and performance.

## Data Availability

Data availability is not applicable to this article as no new data were created or analyzed in this study.
